# Addressing knowledge gaps in Parkinson’s disease: a report on the Movement Disorder Society’s Centre-to-Centre initiative to improve Parkinson’s disease services in Lao People’s Democratic Republic

**DOI:** 10.1186/s12909-020-02161-x

**Published:** 2020-07-29

**Authors:** Onanong Phokaewvarangkul, Somchit Vorachit, Appasone Phoumindr, Saysavath Keosodsay, Ronald B. Postuma, Wassilios G. Meissner, Roongroj Bhidayasiri

**Affiliations:** 1 Chulalongkorn Centre of Excellence for Parkinson’s Disease & Related Disorders, Department of Medicine, Faculty of Medicine, Thai Red Cross Society, Chulalongkorn University and King Chulalongkorn Memorial Hospital, Bangkok, 10330 Thailand; 2 University of Health Science, Vientiane, Lao PDR; 3 Department of Neurology, Montreal General Hospital, Montreal, Quebec, Canada; 4 Service de Neurologie, CHU Bordeaux, 33000 Bordeaux, France and Institut des Maladies Neurodégénératives, Université de Bordeaux, UMR 5293, 33000 Bordeaux, France; 5 Department of Medicine, Christchurch, and New Zealand Brain Research Institute, University of Otago, Christchurch, New Zealand

**Keywords:** Education, Parkinson’s disease, Medical professionals, Underserved region, Developing country

## Abstract

**Background:**

Lao People’s Democratic Republic (Lao PDR) has only nine neurologists for seven million people; none have formal training in Parkinson’s disease (PD). Medical specialists require sufficient PD knowledge to provide high-quality care.

**Methods:**

This study outlines a Centre-to-Centre programme for developing PD expertise in underserved regions through a tailored two-year educational enterprise between an established movement disorder mentor centre at Chulalongkorn University in Thailand and mentee centres in Lao PDR. Background knowledge of 80 Laotian physicians was assessed using a validated PD knowledge questionnaire containing 26 questions divided into 3 sections (diagnosis, therapeutic options, disease course) before and immediately after one-day kick-start training. Responses were compared across physicians’ demographic groups.

**Results:**

Of 80 respondents, 50 (62.5%) were board-certified physicians, of which 27 (54%) specialised in internal medicine. Apparent knowledge gaps were shown by a 51.2% correct response rate for total score, 52.8% for diagnosis, 50.6% for therapeutic options, and 48.2% for disease course. No significant differences in total score or any domain sub-scores between neurologists and other specialties were found. Many did not know which non-motor symptoms could occur as prodromal symptoms or late in course of PD. Incorrect responses mainly reflected a lack of knowledge of the impact of medication on disease. Total and domain sub-scores significantly improved after the course (*p* < 0.05, each). The size of difference of the means was significant for the total score (d = 0.82), therapeutic option (d = 0.56), and disease course (d = 0.68) sub-scores.

**Conclusions:**

Significant improvement of PD knowledge amongst Laotian physicians is demonstrated after a training course, focusing on practical management of PD. Our findings highlight the importance of continued medical education, especially PD-specific training.

## Background

Parkinson’s disease (PD) represents a global public health issue, with millions of affected patients worldwide. However, many regions around the world still lack sufficient resources to provide adequate care to PD patients [1, 2]. Moreover, a lack of satisfactory knowledge amongst medical specialists may present a barrier for PD patients to access suitable treatments, particularly in developing nations [3, 4], where PD knowledge is often insufficient [4]. Although the World Medical Assembly endorses and promotes patient autonomy and access to good quality medical care without discrimination, following the Declaration of Lisbon on patients’ rights in 1984, there are notable challenges in providing equitable health care in developing countries with ageing populations, further highlighting healthcare inadequacy in underdeveloped countries [5]. For instance, in Sub-Saharan Africa (SSA), the lack of case studies of PD led to a belief that PD was less common in SSA than the rest of the world. However, current evidence suggests it is actually that PD patients in SSA have poor access to healthcare and therefore are not identified [6]. Even if diagnosed, patients rarely take medications as they are not widely available or subsidised by healthcare system [7]. It is likely that the situation in SSA is replicated in other developing countries, including Lao People’s Democratic Republic (Lao PDR), a developing country in Southeast Asia [1]; this situation in Lao PDR would then exemplify the limited treatment accessibility and resources for many neurological disorders in this region [8, 9].

In Lao PDR, there are only nine neurologists for seven million people, with the vast majority of them living in the capital city of Vientiane. Knowledge of common neurological disorders amongst both the Laotian people and Laotian medical trainees is only beginning to be formalised [9]. Although medical education and curricula in Lao PDR has included subjects related to neurological diseases, such as epilepsy and stroke, PD and other movement disorders are not officially listed. Initiatives to improve treatment for epilepsy in Lao PDR have been available since 2011. This has led to dedicated epilepsy services within the country, including a specialty clinic for epilepsy and the availability of electroencephalography [10–12]. A similar initiative was later developed for stroke care to include a stroke referral system and stroke fast track programme in 2015 [10, 11]. Although PD is a common neurodegenerative disease amongst the elderly, with a substantial increasing trend in the number of patients [13], there is no existing collaboration network between Thailand and Lao PDR. Despite educational collaboration partnerships between Chulalongkorn University and the University of Health Sciences, none of the Laotian neurologists possess formal training in PD, and there is neither formal PD educational training nor any established instrumental media, e-learning, or telemedicine programmes to address this lack. Furthermore, only two anti-parkinsonian medications are available in Lao PDR: levodopa and trihexyphenidyl (Additional file 1) [14].

The Chulalongkorn Centre of Excellence for PD and Related Disorders (ChulaPD; www.chulapd.org) has become a mentor centre for delivering training and cultivating growth of mentee centres in Laos. Mentorship is provided through a two-year Centre-to-Centre (CTC) pilot programme funded by the International Parkinson and Movement Disorders Society (MDS). The CTC action plan for Thailand and Laos is summarised in Additional file 2. The main goal of the MDS CTC programme is to enhance PD treatment accessibility in underserved regions worldwide. Sufficient knowledge about symptoms and disease progression and enhanced community awareness are vital to increase the chance of a correct diagnosis and the delivery of high-quality care to PD patients. In addition, increased availability of suitable investigations and medication should be promoted. The programme also aims to establish the most suitable content for continuing medical education curricula and to increase awareness of the factors that contribute to the medical community’s knowledge gaps. We report on the first steps of the two-year CTC programme which was to assess knowledge gaps related to PD in Laotian medical providers. We also evaluated the effect of a one-day targeted educational programme to improve the level of PD knowledge among this group.

## Methods

### Participants and study design

This study is a cross-sectional, validated questionnaire-based study to ascertain the level of PD medical knowledge amongst Laotian medical professionals. Participants included 80 physicians, currently licensed to practice in Laos, who volunteered to attend a short course on PD and movement disorders in Vientiane in July 2019. All participants belonged to an existing network group for stroke and epilepsy at the University of Health Science, Vientiane. Demographic information was collected from all participants. These physicians treat neurological patients. Participants were grouped as board-certified physicians or medical residents. Board-certified physicians had received three or more years of additional formal training and obtained certificates in certain specialties, such as neurology, general medicine, and psychiatry (Table 1), while medical residents included physicians who graduated with a Doctor of Medicine (MD) degree, were licensed to practice in Lao PDR, and were enrolled in board-certified training programmes from the University of Health Science located in Vientiane. All participants either work as doctors employed by the government or university or have a private practice.

**Table 1:** Demographic data and results of all Laotian physicians

Items	Medical physicians (*n* = 80)	Board-certified physicians (*n* = 50)	Medical residents (*n* = 30)	*p*-value
Age	33.75 ± 6.91	35.32 ± 7.83	31.13 ± 3.92	0.01*
Male gender	34 (42.5%)	22 (44%)	12 (40%)	0.73
Years of medical practice	7.55 ± 5.37	8.36 ± 6.20	6.20 ± 3.24	0.04*
Pre-evaluation				
• Total score (26 points)	13.31 ± 3.09	14.30 ± 2.92	12.50 ± 2.43	0.01*
• Diagnosis section (13 points)	6.87 ± 1.79	7.34 ± 1.68	6.57 ± 1.55	0.04*
• Therapeutic options section (7 points)	3.54 ± 1.21	3.88 ± 1.24	3.20 ± 0.96	0.01*
• Disease course section (6 points)	2.89 ± 1.21	3.08 ± 1.19	2.73 ± 1.20	0.21
Post-evaluation				
• Total score (26 points)	15.61 ± 2.27	15.77 ± 2.52	15.37 ± 1.88	0.52
• Diagnosis section (13 points)	7.69 ± 1.74	7.74 ± 1.82	7.62 ± 1.66	0.80
• Therapeutic options section (7 points)	4.27 ± 1.03	4.49 ± 0.89	3.96 ± 1.16	0.06
• Disease course section (6 points)	3.64 ± 0.98	3.54 ± 1.04	3.79 ± 0.88	0.34

Statistical analysis of data between board-certified physicians group and medical residents group was performed by independent sample *t*-test or Chi-square test; mean ± SD represented for mean and standard deviation; N: number of patients; *: denotes statistical significant results

### The Centre-to-Centre programme

The 2-year CTC programme was a collaborative project between ChulaPD (www.chulapd.org) as the mentor centre and the University of Health Science as the mentee centre, with funding support from the MDS. The mentor centre was responsible for creating and delivering appropriate PD educational training based on the mentees’ interests and knowledge gaps. Regular tele-education and in-person visits between mentor and mentee centres provided core PD teaching based on our proposed knowledge-based concept (Additional file 3), which focused on strengthening medical professionals’ theory and practice knowledge. The programme was approved by relevant MDS stakeholders including the MDS International Education Committee.

### Preparatory field experience and setting of a one-day teaching course

We identified that only three of the four major governmental hospitals in Vientiane had full-time neurologists, none of whom had received specific PD training. In addition, outdated instruments based on old textbooks were used in the educational PD programmes for Laotian general practitioners. Therefore, a one-day PD and movement disorders educational course was offered to Laotian medical professionals in July 2019. Detailed activities are provided in Additional file 5. We conducted pre- and post-course evaluations to identify participants’ background and after course knowledge using a validated PD questionnaire [4]. The full agenda is presented in Additional file 4.

### PD knowledge questionnaire

A 26-item, three-domain, true or false questionnaire was created to investigate PD knowledge [4]. The three domains included diagnosis (13 items; total score 13), therapeutic options (7 items; total score 7), and disease course (6 items; total score 6). Higher scores indicated a higher level of PD knowledge. The questionnaire was devised, validated, and made available in Thai and English [4, 15], since the Thai and Laotian languages are highly similar and use the same lexicon, both being derivatives of the same ancestor tongue. A total of 15 min was given to complete the questionnaire, which was administered and collected just prior to and immediately after the training course. The questionnaire format was either paper-based or used a self-completed Google document. The full questionnaire is provided in Additional file 6. The study was approved by the Human Ethics Committee of the Faculty of Medicine, Chulalongkorn University, and the Human Ethics Committee of the Faculty of Medicine, University of Health Science. All participants gave informed consent before entering the study, in accordance with the Declaration of Helsinki.

### Data analysis

Pre- and post-test evaluation amongst normal distribution were analysed using chi-squared tests for categorical data and paired-t test for continuous data. Pearson’s correlation coefficients evaluated the correlations between variables. A *p*-value < 0.05 was regarded as statistically significant. Although a *p*-value could inform whether an effect of difference existed, it could not reveal the magnitude of effect as an effect size [16]. An effect size for mean difference of the total PD knowledge and each sub-score was calculated for Cohen’s *d*. Stepwise linear regression was conducted to identify the variables most likely to predict a high level of PD knowledge in individual physicians. The Durbin–Watson test was used to identify an auto-correction in the regression analysis, where a value close to 2 indicates no first-order serial correlation. IBM SPSS Statistics, version 23.0 (IBM, Armonk, NY) was used for all statistical analyses.

## Results

Table 1 shows participants’ demographic and clinical characteristics. Of 80 respondents, 50 (62.5%) were board-certified physicians and 34 (42.5%) were men. Of the 50 board-certified physicians, 27 (54%) were certified in internal medicine, 9 (18%) in psychiatry, 8 (16%) in neurology, and 6 (12%) in other specialties. The other 30 (37.5%) were medical residents. The mean age of all respondents was 33.75 years (SD 6.91); medical residents were much younger on average than board-certified physicians (*p* = 0.01), and had significantly fewer years of experience in medical practice (*p* = 0.04). Twenty participants (25%) had obtained their last professional credentials at least 10 years previously.

**Fig. 1:**
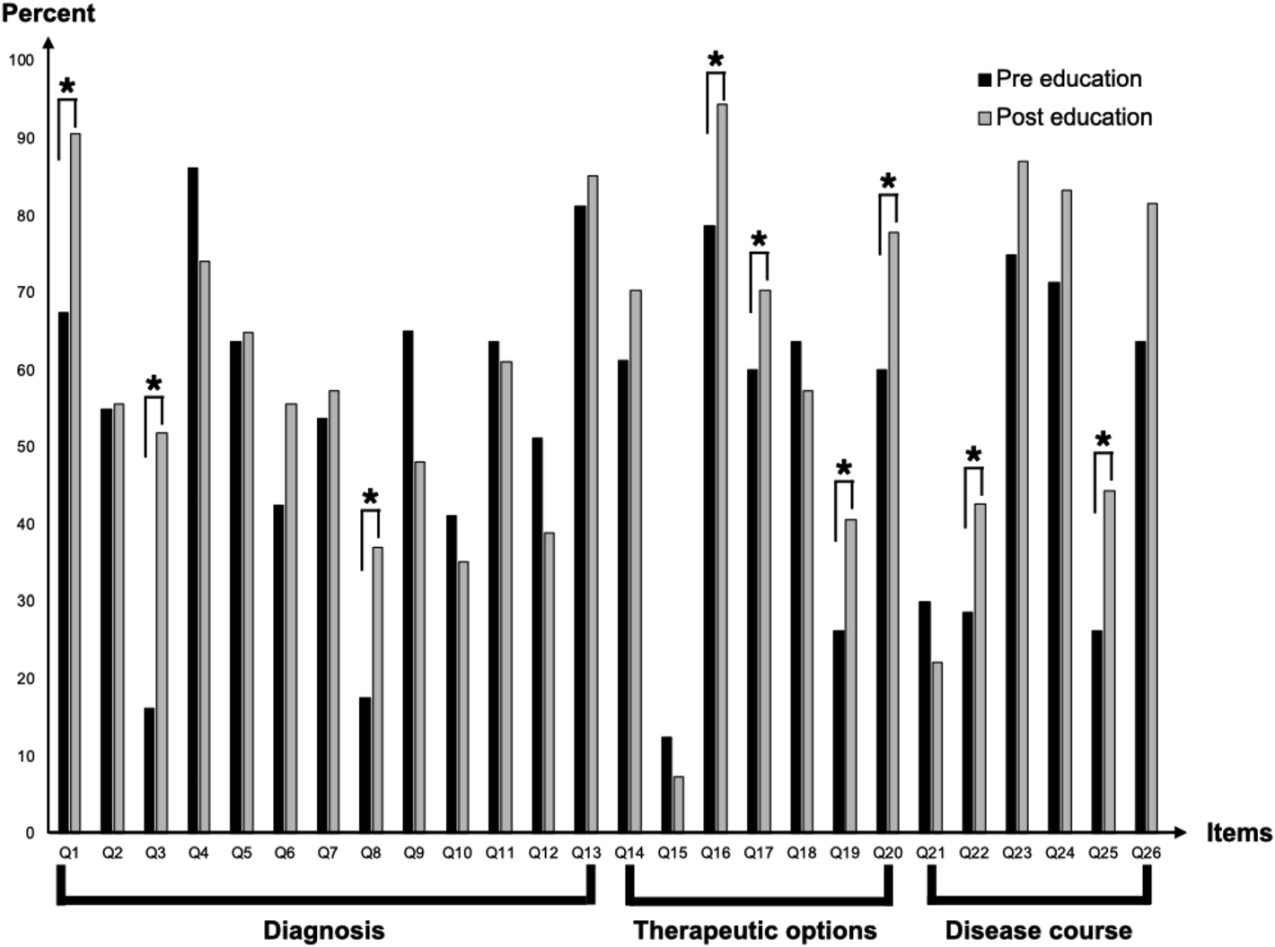
Percentage of correct responses on individual items between pre- and post-course evaluation. In the diagnosis domain, significant improvement of correct responses was observed in the following items; Q1: definite diagnosis of PD requires neuro-imaging confirmation; Q3: the presence of rest tremor is mandatory for the diagnosis of PD; and Q8: freezing of gait often precedes tremor and/or bradykinesia and dyskinesia (Q8). In the therapeutic options domain, significant improvement of correct responses were observed in the following items: Q16) the most effective drug available for PD is levodopa; Q17: levodopa has been shown to accelerate the progression of PD; Q19: dopamine agonists have definite evidence on slowing disease progression; and Q20: PD can be cured by deep brain stimulation surgery. In the disease course domain, significant improvement of correct responses was observed in the following items: Q22: wheelchair- or bed-bound is inevitable in PD; and Q25: deep brain stimulation surgery can stop PD progression

The total score revealed numerous incorrect responses about PD amongst Laotian medical professionals, with an approximately 50% correct response rate for each item—total score (51.2%), diagnosis (52.8%), therapeutic options (50.6%), and disease course (48.2%) (Table 1). Medical residents had a significantly lower number of correct responses than board-certified physicians, primarily in the diagnosis and therapeutic domains (Table 1). Pre- and post-course PD knowledge evaluations are shown in Table 2. For all physicians, significant improvement in PD knowledge was demonstrated in the total score and all domains sub-scores (*p* < 0.01, each). Additionally, the effect size for the total score was large (Cohen’s d of 0.82, 95% CI [0.46, 1.19]), indicating an apparent magnitude of difference between pre- and post-course outcomes. Amongst all three domains, the effect sizes for the therapeutic options and disease course sub-scores were medium (Cohen’s d of 0.56, 95% CI [0.20, 0.99] and 0.68, 95% CI [0.32, 1.04] respectively) whereas the effect size for the diagnosis sub-score was small (Cohen’s d of 0.42, 95% CI [0.07, 0.77]). Details of individual item improvement were shown in Fig. 1. Following the training, all board-certified physicians demonstrated a significant improvement in PD knowledge in the total score as well as therapeutic options, and disease course sub-scores (*p* < 0.01, each), but not in the diagnosis sub-score (*p* = 0.14). Significant improvement of PD knowledge amongst medical residents was shown in the total score and all three domains sub-scores (*p* < 0.05, each) (Table 2).

**Table 2:** Results of pre- and post-course PD knowledge evaluations of all Laotian physicians

Items	All physicians (*N* = 80)		Board-certified physicians (*N* = 50)		Medical residents (*N* = 30)		*p*-value
	Pre	Post	Pre	Post	Pre	Post	
• Total score	13.31 ± 3.09	15.61 ± 2.27	14.30 ± 2.92	15.77 ± 2.52	12.50 ± 2.43	15.37 ± 1.88	*p*_*A*_ < .001*
							*p*_*B*_ < .001*
							*p*_*M*_ < .01*
• Diagnosis section	6.87 ± 1.79	7.69 ± 1.74	7.34 ± 1.68	7.74 ± 1.82	6.57 ± 1.55	7.62 ± 1.66	*p*_*A*_ < .01*
							*p*_*B*_ = 0.14
							*p*_*M*_ < .05*
• Therapeutic options section	3.54 ± 1.21	4.27 ± 1.03	3.88 ± 1.24	4.49 ± 0.89	3.20 ± 0.96	3.96 ± 1.16	*p*_*A*_ < .001*
							*p*_*B*_ < .01*
							*p*_*M*_ < .01*
• Disease course section	2.89 ± 1.21	3.64 ± 0.98	3.08 ± 1.19	3.54 ± 1.04	2.73 ± 1.20	3.79 ± 0.88	*p*_*A*_ < .001*
							*p*_*B*_ < .01*
							*p*_*M*_ < .001*

Statistical analysis of data between pre- and post-evaluation amongst board-certified physicians group and medical residents group was performed by paired sample *t*-test, which *p*_*A*_ represents for all physicians, *p*_B_ represents for board-certified physicians group and *p*_M_ represents for medical residents group. Mean ± SD represented for mean and standard deviation; N: number of patients; *: denotes statistical significant results

No significant correlations were found between physician’s age and PD knowledge: diagnosis (*r* = 0.08, *p* = 0.46), therapeutic (*r* = − 0.06, *p* = 0.62), disease course (*r* = 0.09, *p* = 0.39), and total score (*r* = 0.05, *p* = 0.65). Further, no noteworthy relationships were apparent between years of medical practice experience and PD knowledge: diagnosis (*r* = 0.03, *p* = 0.81), therapeutic (*r* = − 0.11, *p* = 0.31), disease course (*r* = 0.17, *p* = 0.14), and total score (*r* = 0.03, *p* = 0.79). Linear regression analysis identified only being a board-certified physician as predictive of an elevated degree of PD knowledge.

## Discussion

This initial report on the CTC programme is the first study to identify and focus on the core knowledge on PD symptoms, diagnosis, and treatment in the Laotian medical community. This current study found the number of Laotian medical specialists with knowledge gaps in PD was higher compared to a previous study of Thai medical professionals [4], with a lower PD knowledge score in every domain as well as incorrect individual item responses, particularly items on PD diagnosis and treatment. In the diagnosis domain, many respondents were unaware of non-motor symptoms (NMS) and doubtful about which types of NMS could occur as prodromal or late PD manifestations, even though a majority had satisfactory knowledge regarding specific motor indicators such as bradykinesia. Responses to the therapeutic items showed that most participants understood the effectiveness of PD treatments such as levodopa, dopamine agonists, and DBS. However, they had knowledge gaps on the effects of medication for reducing disease progression, particularly dopamine agonists and shared certain mistakes about PD related disabilities, DBS, and the link between PD subtypes and disease progression.

In both the diagnosis and therapeutic domains, medical residents had a higher number of incorrect responses and lower PD knowledge scores than the board-certified physicians, but similar low scores in the disease course domain. These results suggested that linking general PD knowledge with Laotian medical education standards to boost the general level of knowledge via continuing medical education may enhance healthcare delivery to PD patients [17, 18]. In order to improve PD knowledge amongst Laotian medical professionals further, medical education on PD should be continuous and focused.

Our results highlight the possibility for targeted education in specific areas of PD in certain groups of Laotian medical professionals. For example, PD-specific training on diagnosis and treatment should be offered to medical residents so they are aware of different classes of dopaminergic medications as well as their therapeutic evidence. However, as part of the Red Cross society network, we are hopeful that more anti-Parkinsonian medications will become available within Lao PDR; the need to educate physicians in this area is key.

Limitations of our study include that the study sample may not accurately represent all Laotian board-certified physicians, since participants practiced a variety of sub-specialties and board-certified physicians in the sample were younger on average than those in the general population. However, the advantage of including these younger board-certified physicians is that they will become the next generation of physicians to provide future healthcare services in Laotian communities. Second, assessing physicians who were unfamiliar with the Thai language may have been more difficult than anticipated. However, a local bilingual neurologist was present to clarify questionnaire items at the local site. Third, no information was available on the prior clinical experience on PD management of the individual participants which could influence their pre-existing knowledge. Fourth, the limited number of items of PD knowledge questionnaire could potentially underestimate the significance of results. Finally, the concepts evaluated in this study did not include physicians’ personal viewpoints and resources; only PD core diagnosis and management were assessed, which may not reflect physicians’ real-life clinical practice.

## Conclusions

This research identified knowledge gaps among Laotian physicians in all aspects of PD, including diagnosis, therapeutic options, and disease course. The findings suggest that PD-focused training needs to be an essential aspect of continuing medical education in Lao PDR.

## Supplementary information

**Additional file 1.** Drug availability across regions and countries. Table represents the number of neurologists across regions and countries and their available drug lists. United States and European countries have all anti-Parkinsonian medications available according to the current practice guideline, whereas countries in the Southeast Asian region have limited drugs, especially in Lao PDR.

**Additional file 2. **The Thailand and Lao PDR Centre-to-Centre programme concept. The Thailand and Lao PDR concept of Centre-to-Centre programme**.** The 2-year Centre-to-Centre (CTC) programme was a collaborative project between Chulalongkorn Centre of Excellence for Parkinson’s Disease & Related Disorders (ChulaPD, www.chulapd.org), as the mentor centre and the University of Health Science as the mentee centre, receiving funding support from the International Parkinson and Movement Disorder Society (MDS). The concept of CTC programme is to establish the movement disorders service in the underserved region by offering a tailored two-year educational training between established “mentor” movement disorder centres and “mentee” centres. Either increasing capacities of local physicians to handle with care for their PD patients, changing the way their practices, or establishing the dedicated movement disorders services are ultimate goals of this project. The responsibilities of the mentor centre are to create and deliver proper educational training about PD based on the mentees’ interest and to identify knowledge gaps which may intimate appropriate educational engagements. A number of regular tele-education and in-person visits between mentor and mentee centres provide core knowledge in PD. In particular, the educational programme will be focused on strengthening both theory and practice knowledge to medical professionals. This programme was approved by the MDS education committee.

**Additional file 3.** Proposed knowledge-based concepts for medical education in underserved regions. Diagram demonstrating the relationship between ‘no knowledge’ until ‘knowledge in theory and practice’. No knowledge develops from no experience, no study, no resources, ignorance, and/or illusory knowledge. After learning, knowledge in theory will be developed. After repetitive exposure to clinical experience and case-based materials, both knowledge in theory and in practice will be constructed.

**Additional file 4.** The agenda of Parkinson’s disease and movement disorder educational programme.

**Additional file 5.** Activity photo of the 1st Centre-to-Centre (CTC) Thailand – Lao PDR Programme on Parkinson’s Disease and Other Movement Disorders. Figure A represents mentor and mentee teams of the CTC Programme; Figure B represents the first collaboration between Chulalongkorn Centre of Excellence for Parkinson’s Disease and Related Disorders (ChulaPD) and the University of Health Science; Figure C shows the conference room, with physicians making up the majority of attendees; Figure D shows outpatient clinic activities during the visit from ChulaPD staff.

**Additional file 6.** Parkinson’s Disease Knowledge Questionnaire.

## Data Availability

The datasets during and/or analysed during the current study available from the corresponding author on reasonable request.

## Abbreviations

ChulaPD: Chulalongkorn Centre of Excellence for PD and Related Disorders
CTC: Centre-to-Centre
DBS: Deep brain stimulation
Lao PDR: Lao People’s Democratic Republic
MD: Doctor of Medicine
MDS: The International Parkinson and Movement Disorders Society
NMS: Non-motor symptoms
PD: Parkinson’s disease
SSA: Sub-Saharan Africa
