# Supplementary figures and images for: Addressing knowledge gaps in Parkinson’s disease: a report on the Movement Disorder Society’s Centre-to-Centre initiative to improve Parkinson’s disease services in Lao People’s Democratic Republic
Source: BMC Med Educ. 2020 Jul 29;20:239. doi: 10.1186/s12909-020-02161-x (PMC7392705; doi:10.1186/s12909-020-02161-x)

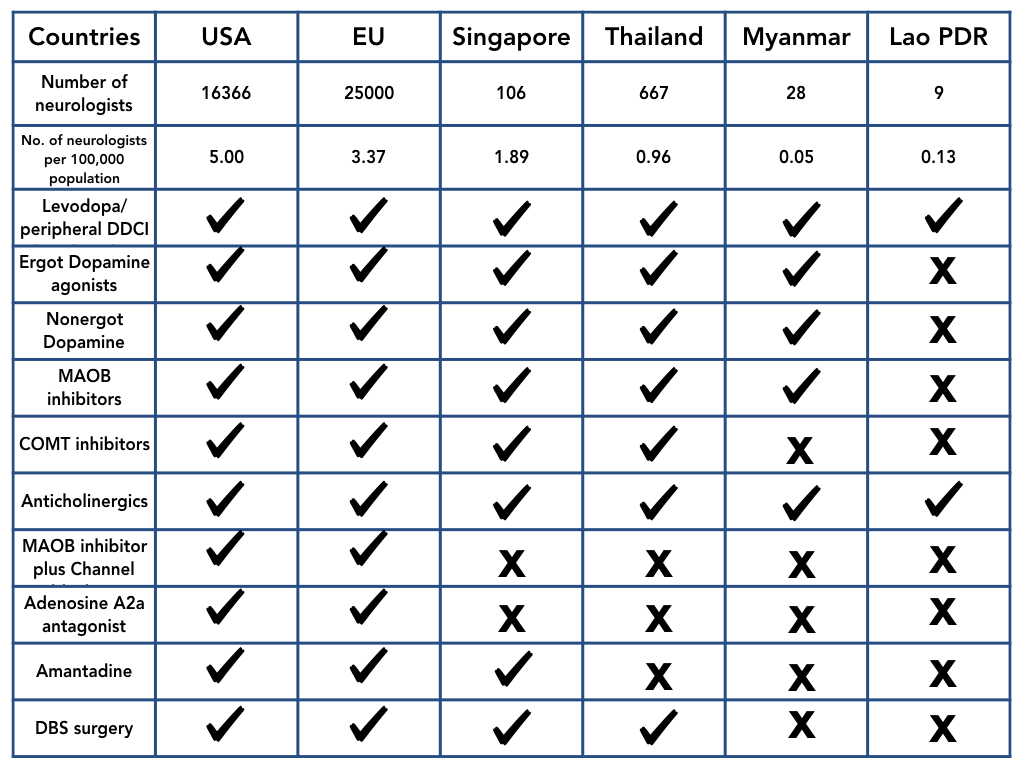

Supplement: Supplementary file 1 — Additional file 1. Drug availability across regions and countries. Table represents the number of neurologists across regions and countries and their available drug lists. United States and European countries have all anti-Parkinsonian medications available according to the current practice guideline, whereas countries in the Southeast Asian region have limited drugs, especially in Lao PDR. [file 12909_2020_2161_MOESM1_ESM.jpeg]

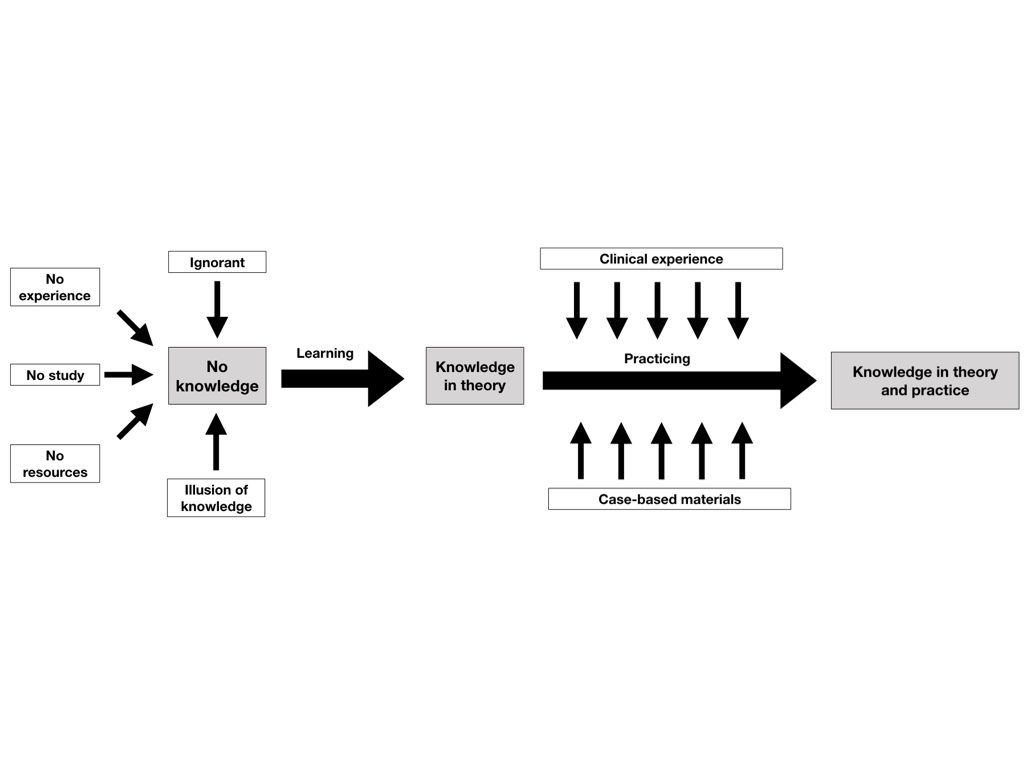

Supplement: Supplementary file 3 — Additional file 3. Proposed knowledge-based concepts for medical education in underserved regions. Diagram demonstrating the relationship between ‘no knowledge’ until ‘knowledge in theory and practice’. No knowledge develops from no experience, no study, no resources, ignorance, and/or illusory knowledge. After learning, knowledge in theory will be developed. After repetitive exposure to clinical experience and case-based materials, both knowledge in theory and in practice will be constructed. [file 12909_2020_2161_MOESM3_ESM.jpeg]

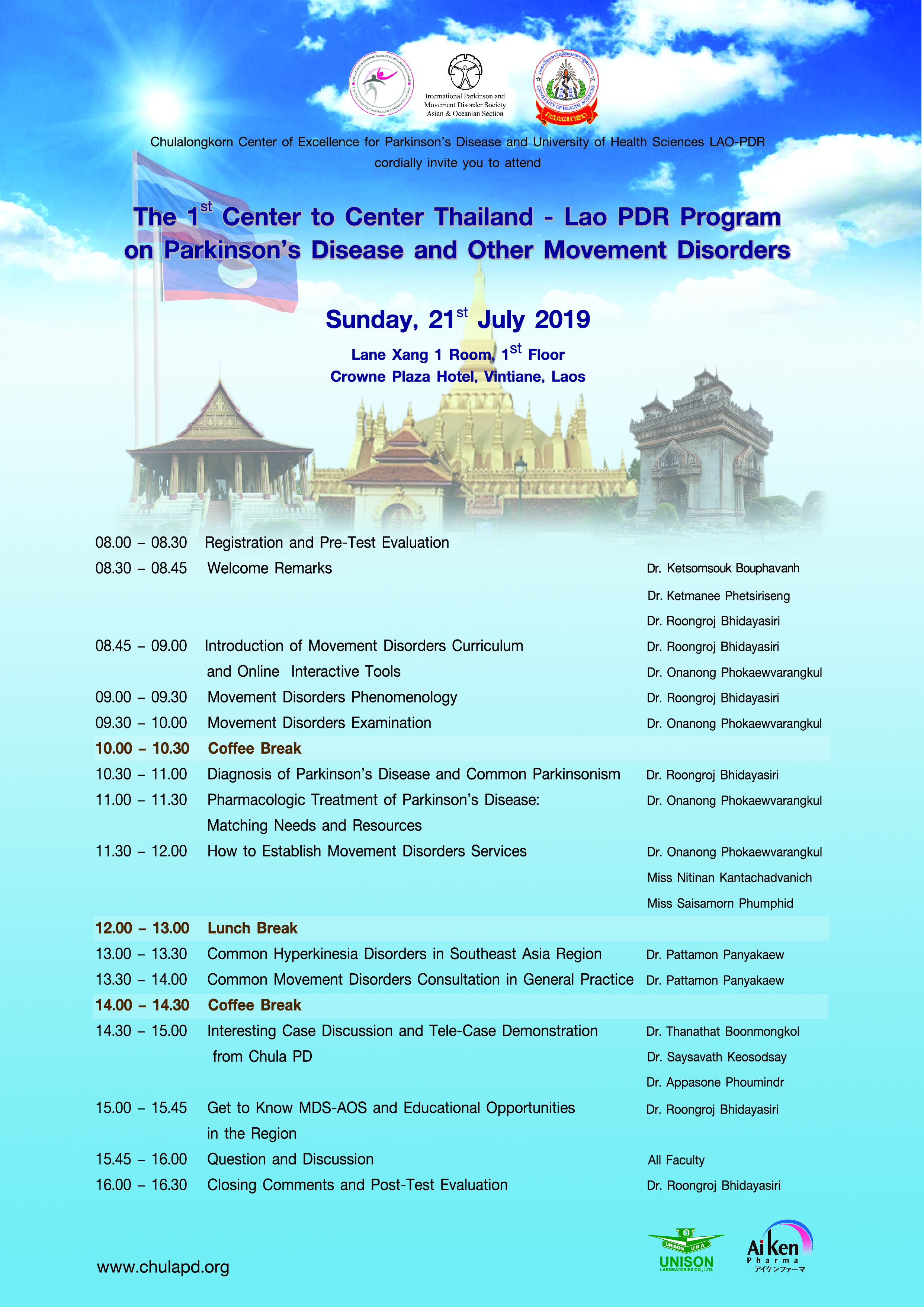

Supplement: Supplementary file 4 — Additional file 4. The agenda of Parkinson’s disease and movement disorder educational programme. [file 12909_2020_2161_MOESM4_ESM.jpg]

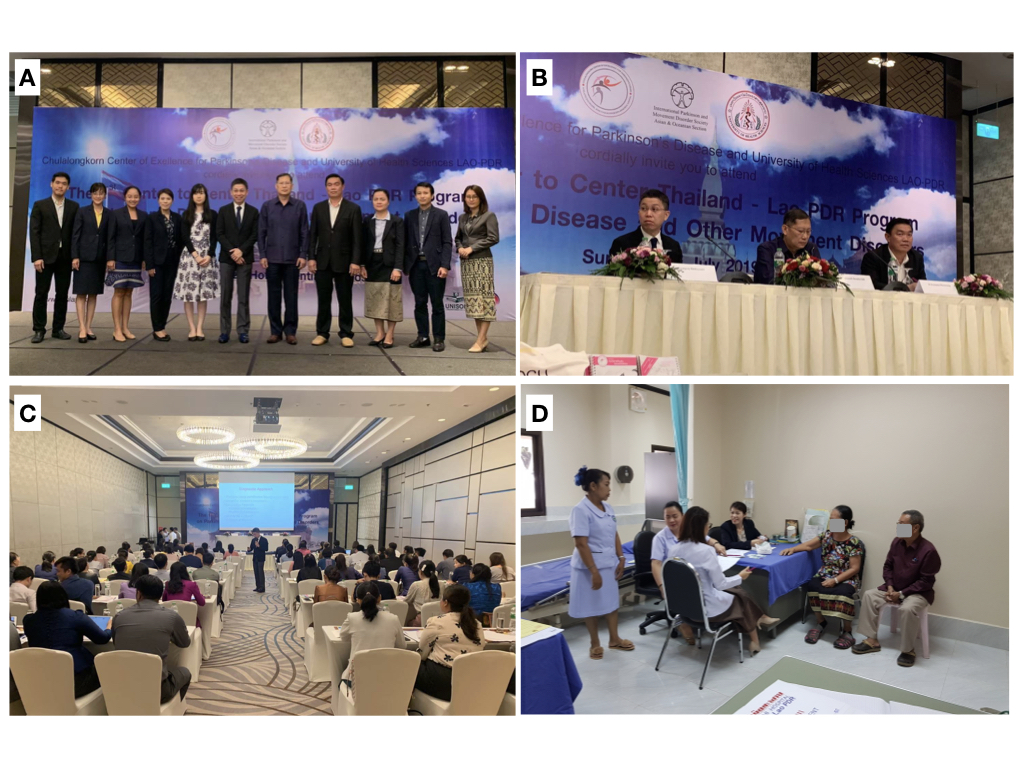

Supplement: Supplementary file 5 — Additional file 5. Activity photo of the 1st Centre-to-Centre (CTC) Thailand – Lao PDR Programme on Parkinson’s Disease and Other Movement Disorders. Figure A represents mentor and mentee teams of the CTC Programme; Figure B represents the first collaboration between Chulalongkorn Centre of Excellence for Parkinson’s Disease and Related Disorders (ChulaPD) and the University of Health Science; Figure C shows the conference room, with physicians making up the majority of attendees; Figure D shows outpatient clinic activities during the visit from ChulaPD staff. [file 12909_2020_2161_MOESM5_ESM.jpeg]
