# Supplementary material for: Addressing knowledge gaps in Parkinson’s disease: a report on the Movement Disorder Society’s Centre-to-Centre initiative to improve Parkinson’s disease services in Lao People’s Democratic Republic
Source: BMC Med Educ. 2020 Jul 29;20:239. doi: 10.1186/s12909-020-02161-x (PMC7392705; doi:10.1186/s12909-020-02161-x)
Supplement: Supplementary file 2 — Additional file 2. The Thailand and Lao PDR Centre-to-Centre programme concept. The Thailand and Lao PDR concept of Centre-to-Centre programme. The 2-year Centre-to-Centre (CTC) programme was a collaborative project between Chulalongkorn Centre of Excellence for Parkinson’s Disease & Related Disorders (ChulaPD, www.chulapd.org), as the mentor centre and the University of Health Science as the mentee centre, receiving funding support from the International Parkinson and Movement Disorder Society (MDS). The concept of CTC programme is to establish the movement disorders service in the underserved region by offering a tailored two-year educational training between established “mentor” movement disorder centres and “mentee” centres. Either increasing capacities of local physicians to handle with care for their PD patients, changing the way their practices, or establishing the dedicated movement disorders services are ultimate goals of this project. The responsibilities of the mentor centre are to create and deliver proper educational training about PD based on the mentees’ interest and to identify knowledge gaps which may intimate appropriate educational engagements. A number of regular tele-education and in-person visits between mentor and mentee centres provide core knowledge in PD. In particular, the educational programme will be focused on strengthening both theory and practice knowledge to medical professionals. This programme was approved by the MDS education committee. [file 12909_2020_2161_MOESM2_ESM.docx]

**The Thailand and Lao PDR** **Concept of Centre-to-Centre programme**

The 2-year Centre-to-Centre(CTC) programme was collaborative project between Chulalongkorn Centre of Excellence for Parkinson's Disease & Related Disorders (Chulapd, [www.chulapd.org](http://www.chulapd.org)), as the mentor centre and the University of Health Science as the mentee centre, receiving funding support from the International Parkinson and Movement Disorder Society (MDS). The concept of CTC programme is to establish the movement disorders service in the underserved region by offering a tailored two-year educational training between partner one established movement disorders “Mentor” centre and “Mentee” centres. Either increasing capacities of local physicians to handle with care for their PD patients, changing the way their practices, or establishing the dedicated movement disorders services are ultimate goals of this project. The responsibilities of the mentor centre are to create and deliver proper educational training about PD based on the mentees interest and their knowledge gaps which may intimate appropriate educational engagements. A number of regular tele-education and in-person visits between mentor and mentee centres provide core knowledge in PD. In particular, the educational programme will be focused on strengthening both theory and practice knowledge to medical professionals. This programme was approved by the MDS education committee.

This project presents the opportunity to extend the mission and vision of the MDS to offer the higher education and skills necessary to endorse growth at mentor centres to mentee centres in underserved regions such as Lao PDR. Implementation of the prepared and suitable educational program will be supported by the mentor center to mentee centers. The advantages for mentee groups are not restrained to the PD-specific educational programme. Prospects for mentee centres and their associated physicians includegaining knowledge about the international movement disorders society, getting applications for membership free of charge, gaining access to MDS educational materials (library, journals, and video) at no charge, and gaining the chance to be present at the yearly MDS conference. Limited resources are an additional concern besides suitable educational programmes. As Lao PDR is one of our bordering nations, the chance to engage in complementary humanitarian support for bordering countries that are disadvantaged in terms of medical services, expertise and supplies is made possible through the Thai Red Cross society. Thus, this programme enhances the opportunities of patients to obtain standard therapeutics throughout the country and creates dedicated movement disorder services with support from mentees in the capital city that will subsidise medical services and resources, which may become critical to enhancing awareness of PD practices, as well as expand PD knowledge among Laotian physicians.
