# Supplementary material for: Addressing knowledge gaps in Parkinson’s disease: a report on the Movement Disorder Society’s Centre-to-Centre initiative to improve Parkinson’s disease services in Lao People’s Democratic Republic
Source: BMC Med Educ. 2020 Jul 29;20:239. doi: 10.1186/s12909-020-02161-x (PMC7392705; doi:10.1186/s12909-020-02161-x)
Supplement: Supplementary file 6 — Additional file 6. Parkinson’s Disease Knowledge Questionnaire. [file 12909_2020_2161_MOESM6_ESM.doc]

**PARKINSON’S DISEASE KNOWLEDGE QUESTIONNAIRE**

Please place an “X” indicating which of the following is true or false:

| **A: Diagnosis of Parkinson's disease (PD)** | **TRUE** | **FALSE** |
| --- | --- | --- |
| 1. Definite diagnosis of PD requires neuro-imaging confirmation |  |  |
| 2. PD only affects patients after 55 years of age |  |  |
| 3. The presence of rest tremor is mandatory for the diagnosis of PD |  |  |
| 4. The presence of bradykinesia is mandatory for the diagnosis of PD |  |  |
| The following symptoms of PD often precede tremors and/or bradykinesia and Dyskinesias |  |  |
| 5. Hypsomia |  |  |
| 6. Dementia |  |  |
| 7. Mood disorders |  |  |
| 8. Freezing of Gait |  |  |
| 9. Constipation |  |  |
| 10. Depression |  |  |
| 11. Sleep disturbances |  |  |
| 12. Both smoking and high caffeine consumption increase the risk of PD |  |  |
| 13. PD diagnosis can be confirmed by the response to a dopaminergic medication |  |  |
| **B: Pharmacological option** | **TRUE** | **FALSE** |
| 14. Almost all patients diagnosed with PD should begin dopaminergic drug as soon as possible |  |  |
| 15. Most of the available drug for PD both treat the symptoms and slow the progression of the disorder |  |  |
| 16. The most effective drug available for PD is levodopa |  |  |
| 17. Levodopa has been shown to accelerate the progression of PD |  |  |
| 18. Dopamine agonists have less risk of motor complications than levodopa |  |  |
| 19. Dopamine agonists have definite evidence on slowing disease progression |  |  |
| 20. PD can be cured by deep brain stimulation surgery |  |  |
| **C: Progression of PD** | **TRUE** | **FALSE** |
| 21. Patients with tremor predominant symptoms progress more rapidly in the degenerative process than those with postural instability and gait problems |  |  |
| 22. Wheelchair-or bed-bound is inevitable in PD |  |  |
| 23. Depression in PD is partly caused by dopamine deficiency |  |  |
| 24. The risk of dementia is greater for those patients with gait and speech disorders |  |  |
| 25. brain stimulation surgery can stop PD progression |  |  |
| 26. Current evidence supports the use of stem cell transplantation as a curative treatment of PD |  |  |
